# A hybrid approach for the analysis of complex categorical data structures: assessment of latent distance learning perception in higher education

**DOI:** 10.1007/s00180-022-01272-x

**Published:** 2022-09-15

**Authors:** Maria Iannario, Alfonso Iodice D’Enza, Rosaria Romano

**Affiliations:** 1 Department of Political Sciences, University of Naples Federico II, Via L. Rodinó, 22, Naples, Italy; 2 Department of Economics and Statistics, University of Naples Federico II, Via Cintia, 21, Naples, Italy

**Keywords:** Distance learning, Location-scale model, Joint data reduction, Recursive partitioning for ordinal data

## Abstract

A long tradition of analysing ordinal response data deals with parametric models, which started with the seminal approach of cumulative models. When data are collected by means of Likert scale survey questions in which several scored items measure one or more latent traits, one of the sore topics is how to deal with the ordered categories. A stacked ensemble (or hybrid) model is introduced in the proposal to tackle the limitations of summing up the items. In particular, multiple items responses are synthesised into a single meta-item, defined via a joint data reduction approach; the meta-item is then modelled according to regression approaches for ordered polytomous variables accounting for potential scaling effects. Finally, a recursive partitioning method yielding trees provides automatic variable selection. The performance of the method is evaluated empirically by using a survey on Distance Learning perception.

## Introduction

Ratings are widely collected and analysed types of data in many scientific fields, such as social and behavioural sciences, public health and medical studies. Examples of these ordinal responses include variables measuring performance (poor, average, excellent), attitude (disagree, neutral, agree), evaluation (not satisfied, neutral, very satisfied), and perception (lowest, average, highest), among others. More than three response alternatives are also generally considered fostering the debate on the optimal number of categories (see, e.g., the seminal papers by Cox III 1980; Preston and Colman 2000).

A vast literature is devoted to the analysis of ordinal responses (see, e.g., McCullagh 1980; Ananth and Kleinbaum 1997; Tutz 2020a, 2012); a comprehensive review is in Agresti (2010). Sometimes multiple items concerning ratings are provided to measure one or more underlying latent constructs (i.e., psychometric scales), leading to more accurate research findings. The analysis is based on the Item Response Theory (IRT) that seeks to model how constructs manifest themselves in terms of observable item responses. Confirmatory factor analysis, and the more general family of structural equation models, provide a powerful method for examining hypothesized relations among a set of measured ordinal variables. The most common method fits the model to polychoric correlations using either weighted least squares (Jöreskog 1994; Muthén 1984) or robust weighted least squares (Muthén et al 2009). The taxonomy of polytomous item response models for ordinal data proposed by Tutz (2020b) is based on exploiting how ordinal models can be devised by using (conditional or unconditional) dichotomisations of response categories.

Alternative approaches assume metric methods coming up the data as on interval scale or summing up the scores of the considered items (see Liddell and Kruschke 2018, for a critical review).

In our contribution, an item reduction analysis is conducted that defines a single meta-item taking into account both: (i) the items association structure; (ii) the heterogeneity characterising the respondents. The item reduction analysis consists of a suitable joint dimension reduction and clustering procedure: the meta-item corresponds to the obtained cluster membership. When the items belong to a unidimensional scale, that is, they measure one common latent trait, the obtained meta-item is, *de facto*, an ‘ordinal response’. Therefore, the meta-item is to be analysed within the framework of cumulative models or other common mixture models introduced for ordinal outcomes. In particular, we consider a regression model for ordered polytomous variables accounting for potential scaling effects to investigate the respondents’ perceptions. A recursive partitioning method yielding two trees is used to select the main variables. The method yields separate trees for the two influential location and scale terms following the strategy in Hothorn et al (2006): the size of each tree is controlled for, according to the significance of the splits. In particular, at each split, tests for cumulative regression models are used: by cutting the trees at non-significant splits, the procedure implicitly selects variables. Alternative classical ordered response models are also examined for completeness. The proposed approach stacks data reduction and modelling, and it is referred to as *hybrid*.

The performance of the method is evaluated empirically with data collected in a 2020 survey aimed to study the impact of Distance Learning (DL) on students’ perception during the Covid-19 pandemic. To investigate the faceted DL impact on students, we examine three different scales proposed and validated in the literature and submitted in the survey: the scale proposed by Amir et al (2020) to study the perspective of DL higher education students, the ‘student stress scale’, proposed and validated by Zurlo et al (2020), and the ‘fear of Covid-19’ scale, proposed by Mahmud et al (2021), that investigates the future career anxiety. In administering the survey, no approval of an ethics committee was needed as it was not a clinical trial, and, therefore, the health of the respondents was not subject to any risk. It is worth noting that no well-known and established theory relates the three scales. Nor is the analysis of the relationship among the different scales the goal of this research. Instead, the central research hypothesis is that DL, measured through a set of items, depends on a series of variables, some of which are attributable to items of psychometric scales, while others correspond to socio-demographic features. Therefore, the first objective is to obtain an optimal synthesis of the DL that also considers the heterogeneity. The meta-item, obtained via the joint data reduction, is a synthesis of the DL perception scale, and it is regressed on a selection of the items from the other scales, and on some demographics.

The remainder of the paper is organised as follows: Sect. 2 dips into the main content of the motivating example; Sect. 3 briefly reports the joint data reduction approaches, whereas Sect. 4 describes how the meta-item is obtained; in Sect. 5 one of the most used approaches to model the obtained ordinal response is reviewed. Some details on the selection of the variables and an alternative competitor to model the dispersion effect are also briefly outlined. Section 6 illustrates the main results and final remarks conclude the paper.

## Motivating example

The Covid-19 pandemic had a major impact on all human activities and education makes no exception. Distance Learning (DL) became the only way to consistently provide an education to students of any age and level. The sudden switch from classroom learning to DL surely had an impact on the students learning experience. The technical setbacks, such as poor internet connection or lack of tools (computers, tablets), are relatively easy to identify, and their effects on the learning process are rather obvious. It is more difficult to study the effects of DL transition on students from a social and psychological perspective. In fact, it is fair to consider the level of adaptation of the students to the DL process as related to the stress for the fear of contagion, the social limitations, and the anxiety for the future career. In order to investigate the faceted DL impact on students, a survey was conducted in 2020 by the Department of Political Sciences, University of Naples Federico II. It refers to 1589 students from 60 Italian Universities, with the University of Naples and University of Bologna being the most represented, with a $25.9\%$ and $18.5\%$ share, respectively. Some results concerning the survey are reported in Iannario et al (2021), Bacci et al (2022), Iodice D’Enza et al (2021), and Iannario et al (2022).

The survey is structured in four item-blocks: the first block contains 19 items on students demographics and their proximity to Covid-19 cases; the second block is of 12 items that measure the DL perception of the students; the third and fourth blocks, respectively with 7 and 5 items, aim at measuring students’ stress and anxiety induced by Covid-19. The aim to study the survey results via a stacked ensemble model motivates our approach. In particular, the idea to synthesise the DL scale and to analyse the drivers of other scales and students demographics prompted the assessment.

Items in the DL scale are reported in Table 1; the scale, as previously mentioned, consists of twelve items on a 4-point Likert scale ranging from 0 (Strongly disagree) to 3 (Strongly agree).

**Table 1:** The distance learning scale

Code	Masurement items
Q1	Clarification sessions are more suitable delivered in distance learning
Q2	Assessment is more suitable delivered in distance learning
Q3	I did not experience any problems during distance learning
Q4	I did not experience stress during distance learning
Q5	I had more time to prepare learning materials before group discussion with distance learning
Q6	I had more time to review all of the learning materials after class with distance learning
Q7	Distance learning gives similar learning satisfaction than classroom learning
Q8	Distance learning could be implemented in the next semester
Q9	Distance learning gives motivation for self-directed learning and eager to prepare learning materials before group discussion
Q10	Communication with lecturers and fellow students is easier with distance learning
Q11	I like distance learning more than classroom learning
Q12	I study more efficiently with distance learning

## From sequential to joint data reduction

To synthesize the students perspective on DL we apply on the DL-related items a joint data reduction approach. Data Reduction (DR, see e.g., Farcomeni and Greco 2016; Markos et al. 2019) is a general definition that encompasses well-established unsupervised learning methods, such as dimension reduction and clustering. In particular, assuming the data structure at hand to be a table with variables on columns and observations on rows, dimension reduction is referred to as column-wise DR: the starting variables are (linearly) combined, and a reduced set of components that preserve most of the original information is obtained. Similarly, clustering methods define a reduced set of prototype objects (centroids), each representative of a group of homogeneous observations; clustering methods can, therefore, be referred to as row-wise DR in that the observations are represented by a reduced set of prototypes.

It is common practice to apply column and row-wise DR one after the other. Such a two-step approach is referred to as tandem analysis, and its application often produces satisfactory results: the dimension reduction removes redundancies and noise from the original data and eases the clustering step.

Consider $\mathbf{X}$ to be a $n\times p$ data matrix where *n* is the number of observations and *p* is the number of continuous variables. Without loss of generality, assume that $\mathbf{X}$ is column-wise centered and that the variables are equally scaled. The first step of the tandem approach consists of a principal component analysis (PCA, Jolliffe 1986). The PCA solution is obtained by optimising the objective function

1 $$\begin{aligned} \min \phi _{\text {PCA}}\left( {\mathbf {A}},{\mathbf {B}}\right) =\left\| {\mathbf {X}} - {\mathbf {A}}{\mathbf {B}}^{\prime } \right\| ^{2}, \end{aligned}$$

where $\left\| \cdot \right\|$ denotes the Frobenius norm, $\mathbf {{A}}=n^{1/2}{\hat{\mathbf{U}}}{\hat{\Sigma }}$ and $\mathbf {{B}}=p^{1/2}{\hat{\mathbf{V}}}$ are the *d*-dimensional row principal coordinates (observations scores) and column standard coordinates (variables scores), respectively. Furthermore, ${\hat{\mathbf{U}}}, {\hat{\mathbf{V}}}$ and ${\hat{\Sigma }}$ contain the first *d* left and right singular vectors, and the *d* largest singular values resulting from the singular value decomposition

2 $$\begin{aligned} n^{-1/2} \mathbf{X} p^{-1/2} = \mathbf{U}{\Sigma }{} \mathbf{V}^{\prime }. \end{aligned}$$

As stated by the Eckart and Young theorem (Eckart and Young 1936), $\mathbf {{A}}\mathbf {{B}}^{\prime }$ represents the best rank *d* approximation of $\mathbf{X}$, in the least squares sense.

In the second step of the tandem analysis, a K-means clustering (MacQueen 1967) procedure is applied on ${\mathbf {A}}$, the observations scores matrix, so that the following objective function is optimised

3 $$\begin{aligned} \min \phi _{\text {KM}}\left( {\mathbf {Z}}_{K}\right) =\left\| {\mathbf {A}} - {\mathbf {Z}}_{K}{\mathbf {G}} \right\| ^{2}, \end{aligned}$$

where $\mathbf{Z}_{K}$ is the indicator coding of the cluster membership, and

$$\begin{aligned} \mathbf{G}=\left( \mathbf{Z}_{K}^{\prime }{} \mathbf{Z}_{K}\right) ^{-1}{} \mathbf{Z}_{K}^{\prime }{} \mathbf{A} \end{aligned}$$

is the cluster centroid matrix. It is clear that the identification of the cluster allocation in step two depends on the low-dimensional scores obtained in step one. On the other hand, the low-dimensional scores are computed irrespective of the underlying cluster structure. As long as most of the variables at hand discriminate among the clusters, the tandem analysis works well; if, instead, there is a subset containing variables that are pairwise correlated on the whole dataset, the dimension reduction step will not preserve the cluster structure, and the tandem analysis fails. This tandem analysis limitation is known in the literature as the *cluster masking* problem, and illustrative examples can be found (see, e.g., Vichi and Kiers 2001).

To overcome the limitations of the tandem analysis, joint DR (JDR) methods seek for a solution that is optimal for both the dimension reduction and the clustering steps: to this end, JDR methods consist of an iterative procedure that alternatively optimise one step given the other. Different JDR methods have been proposed for continuous (De Soete and Carroll 1994; Vichi and Kiers 2001), categorical (Hwang et al 2006) and mixed-type variables (see, van de Velden et al 2019, for a review). The focus is on reduced K-means (RKM, De Soete and Carroll 1994), and on its categorical analogue cluster correspondence analysis (CCA, van de Velden et al 2017).

A classic example of JDR method for continuous variables is RKM. The RKM aims to solve the simultaneous dimension reduction and cluster analysis problem so that both cluster allocation and dimension reduction maximise the *between* variance of the clusters in the reduced space. The RKM objective function is

4 $$\begin{aligned} \min \phi _{\text {RKM}}\left( {\mathbf {B}},{\mathbf {Z}}_{K}\right) =\left\| {\mathbf {X}} - {\mathbf {Z}}_{K}{\mathbf {G}} {\mathbf {B}}^{\prime }\right\| ^{2}. \end{aligned}$$

An iterative alternating least squares procedure is used to obtain both the cluster allocation of the observations and the variable weights.

## Ordinal response via JDR for survey data

In survey data, the item responses are coded as categorical variables, therefore RKM is not suitable. The categorical counterpart of RKM is CCA, which is the method of choice for the JDR of the DL scale at hand. In particular, each DL item is coded as an indicator matrix ${\mathbf {Z}}_{j}$ of size $n\times p_{j}$: each row corresponds to a respondent, and the columns represent the $p_{j}$ levels of agreement for the *j*th item. Note that this is the same coding used for the cluster membership variable, which has *K* levels, and the corresponding indicator is the $n\times K$ matrix $\mathbf{Z}_{K}$. Observed responses are coded by ones and all other elements are zero. Data from multiple items are collected in the block matrix $\mathbf {Z=}\left[ {\mathbf {Z}}_{1},\ldots ,{\mathbf {Z}}_{p}\right]$. The application of CCA on the DL-related item leads to the definition of a cluster membership variable, and the CCA objective is

5 $$\begin{aligned} \min \phi _{\text {CCA}}\left( {\mathbf {B}}^{\star },{\mathbf {Z}}_{K}\right) =\left\| {\mathbf {D}}_{z}^{-1/2}\mathbf {MZ} - {\mathbf {Z}}_{K}{\mathbf {G}} {\mathbf {B}}^{\star \prime }\right\| ^{2} \ \ \text { s.t. } \ \ {\mathbf {B}}^{\star \prime }{\mathbf {B}}^{\star } = {\mathbf {I}}_{d}, \end{aligned}$$

where ${\mathbf {M}}={\mathbf {I}}_{n}-{\mathbf {1}}_{n}{\mathbf {1}}_{n}^{^{\prime }}/n$ is a centring operator, ${\mathbf {B}}^{\star }=\frac{1}{\sqrt{np}}{\mathbf {D}}_{z}^{1/2}{\mathbf {B}}$, ${\mathbf {D}}_{z}=diag\left( {\mathbf {Z}}^{\prime }{\mathbf {Z}}\right)$, ${\mathbf {B}}$ is the item weights matrix.

Comparing Formula (4) and Formula (5), we see that CCA can be defined as an RKM of a centred and standardised indicator matrix. The standardisation operator for categorical variables is the squared root of the margins. Typically, in CCA, the loadings are standardised accordingly. The observations scores are obtained indirectly, according to the obtained variable quantifications, formally

6 $$\begin{aligned} {\varvec{\Psi }}=\sqrt{\frac{n}{p}}{} \mathbf{M}{} \mathbf{Z}{} \mathbf{D}_{z}^{-1/2}{} \mathbf{B}^{{\star }}. \end{aligned}$$

Since it is not possible to minimise the loss function with respect to $\mathbf{B}^{{\star }}$ and $\mathbf{Z}_{K}$ simultaneously, an alternated least squares iterative procedure is used. Given a user-defined *K*, the cluster allocation $\mathbf{Z}_{K}$ is randomly initialised, then the procedure iterates over the following two steps

- for fixed $\mathbf{Z}_{K}$, find $\mathbf{B}^{{\star }}$ that minimises the objective in (5)
- for fixed $\mathbf{B}^{{\star }}$, update $\mathbf{Z}_{K}$ via a K-means on the observations scores ${\varvec{\Psi }}$.

Convergence is guaranteed as the objective function value does not increase from one iteration to the next one. As in *K*-means, however, multiple random starts are needed to limit the risk of local minima.

The meta-item corresponds to the cluster membership, with levels ordered according to the cluster characterisation. To measure the cluster characterisation due to the *j*th item, we consider the $K\times p_{j}$ standardised residual matrix

$$\begin{aligned} {{\mathcal {R}}}_{j}=\mathbf{D}_{K}^{-1/2}\left( \mathbf{Z}_{K}^{\prime }\mathbf{Z}_{j} - \mathbf{c}_{k}{} \mathbf{c}_{j}^{\prime }\right) \mathbf{D}_{z_{j}}^{-1/2} \end{aligned}$$

$\mathbf{c}_{K}=diag(\mathbf{Z}_{K}^{\prime }{} \mathbf{Z}_{K})$ is the cluster-sizes vector, $\mathbf{c}_{j} = diag(\mathbf{Z}_{j}^{\prime }\mathbf{Z}_{j})$ and $\mathbf{D}_{K}=diag(\mathbf{c}_{K})$. The *k*th row of ${{\mathcal {R}}}_{j}$ indicates the deviation of the observed within cluster item frequency distribution from the distribution in the case of independence. In other words, if the frequency distribution of the item *j* is the same within each cluster, the item *j* and the cluster membership variable are independent, and the corresponding ${{\mathcal {R}}}_{j}$ is filled with 0’s. On the contrary, if the *l*th level of item *j* is particularly frequent (or, infrequent) then ${r}_{kl}$ is high (in absolute value), and the item level in question highly characterise the *k*th cluster.

## Models for ordinal response

The obtained meta-item is a synthesis of the DL perception, and it is referred to as *Y*, an ordinal variable with *K* levels. One of the candidate models to analyse *Y* is the *ordinal regression model*. The different ways to compare the categories of *Y* correspond to cumulative models, adjacent categories and sequential models. The taxonomy given in Tutz (2020a) consists of conditional and non-conditional models, depending on the binary models contained in the ordinal structure. The cumulative is the only non-conditional model that does not use conditioning in its binary building blocks. However, the model parametrisation focuses on location only, ignoring potential heterogeneity in the population. Therefore, we consider the proposal by McCullagh (1980), that takes into account the possible presence of heterogeneity: it has been demonstrated that misleading effects can occur if one ignores the presence of a scaling component. Note that, in our approach, the heterogeneity may be partially disclosed by the JDR step. The location-scale model—also known as the heterogeneous choice or heteroscedastic logit model—has been implemented and extended by several authors (e.g., Cox 1995; Tutz and Berger 2017, 2021; Ishwaran and Gatsonis 2000; Hedeker et al 2008, 2009, 2012, among others). The way to introduce variance heterogeneity is to model it explicitly as a function of the variables. The general idea of the location-scale model is that a latent continuous variable $Y^{\star }$ underlies the ordinal response *Y*, and the model has the form

7 $$\begin{aligned} {Y}^{{\star }}_{i} = \mathbf{X}_{i}{\varvec{\beta }}+\sigma _{i}{\varvec{\epsilon }}_{i}, \end{aligned}$$

where $\varvec{\beta }$ is the *s*-dimensional vector of coefficients, $\mathbf{X}_{i}$ is the row vector of the matrix $\varvec{X}$ which includes *s* variables for the *i*th subject, and $\epsilon _{i}$ is the error term. In the model, $\sigma _{i}$ is the standard deviation of the noise variable ${\varvec{\epsilon }}_{i}$ whose distribution function is *F*(.). Hence F^{−1}^(.) defines the link function. The most common choices for *F*^{−1}^(.) are the logit and probit links (based on logistic and standard normal distributions, respectively), but minimum and maximum extreme value, distributions may also be used. The latter are based on the Gumbel distribution which is positively skewed, for the distribution of the maxima, and the complementary loglog model which is the mirror distribution for the minima. Peyhardi et al (2016) gave a careful investigation of the relationship among ordinal models with different link functions and derived invariance properties for the models. We assume, for the sake of simplicity, the logit link only.

The effects of the variables on the variances are modelled as follows:

8 $$\begin{aligned} \sigma _{i}=exp\left( \mathbf{T}_{i}{\varvec{\eta }}\right) \end{aligned}$$

where $\varvec{T}_{i}$ is the additional vector of $s^{\star }$ variables that impact on the scale and $\varvec{\eta }$ the corresponding coefficients vector.

Since *Y* is a categorised version of $Y^{\star }$, it results that

$$\begin{aligned} \tau _{j-1} < Y^{\star }_{i} \le \tau _{j} \Longleftrightarrow Y_{i}=j; \qquad \qquad j=1,2,\ldots ,K, \end{aligned}$$

where $-\infty =\tau _0< \tau _1<\ldots < \tau _{K}=+\infty$ are the thresholds of $Y^\star$. Simple derivation yields that the response probabilities are given by

$$\begin{aligned} Pr\left( {Y_i \le j\mid {\varvec{X}}_{i}, {\varvec{T}}_{i}}\right) =F\left( \frac{\tau _{j}-{\varvec{X}}_{i}{\varvec{\beta }}}{\exp ({\varvec{T}}_{i}{\varvec{\eta }})}\right) \end{aligned}$$

that, with the logistic distribution, becomes

$$\begin{aligned} log\left( \frac{Pr\left( {Y_i \le j\mid {\varvec{X}}_{i}, {\varvec{T}}_{i}}\right) }{Pr\left( {Y_i > j\mid {\varvec{X}}_{i}, {\varvec{T}}_{i}}\right) }\right) = \frac{\tau _{j}-{\varvec{X}}_{i}{\varvec{\beta }}}{\exp ({\varvec{T}}_{i}{\varvec{\eta }})}. \end{aligned}$$

According to the model, two terms specify the impact of variables: the location term $\tau _{j}-{\varvec{X}}_{i}{\varvec{\beta }}$, and the variance or scaling term $\exp (\varvec{T}_i\varvec{\eta })$, which derives from Eq. (8).

If ${\varvec{X}}_{i}$ and ${\varvec{T}}_{i}$ are different, the interpretation of the ${\varvec{X}}$-variables is the same as in the cumulative models yielding the proportional odds assumption, which implies that the effect of a change in the variables does not depend on the response category, i.e. the $\varvec{\beta }$ are constant with respect to *j* (Agresti 2010).

Inference for these models is based on the likelihood function, whose expression can be found in McCullagh (1980). The likelihood function is maximised via iterative least squares estimation methods (see Tutz 2012, for details). The global validation of the fitted model is performed according to both likelihood-based methods and descriptive measures (see, e.g.,Veall and Zimmermann 1996). The selection of the best model is obtained by comparing Likelihood-ratio tests (LR-test) for nested models and information criteria for non-nested ones. Among information criteria, the Bayesian Information Criterion (BIC) (Schwarz 1978) provides the most parsimonious solution.

### Tree-structured location-scale models

The selection variables method has been pursued by means of a tree-structured approach, as proposed by Tutz and Berger (2021); the approach in question is different from the model-based recursive partitioning implemented by Zeileis et al (2008).

In particular, two separated trees are trained for the location and scale terms. Following the strategy proposed by Hothorn et al (2006), tests for cumulative regression models are used to select the single splits, and, consequently, the location and scale trees. While a detailed description of the strategy is beyond the scope of the paper, here follows an intuition. Let $T_{s(c_{s})}$ be the likelihood-ratio (LR) test statistics for variable *s* and the split point $c_s$; the maximal value statistic is found such that $T_s = max_{c_s}T_{s(c_{s})}$, considering all the possible splits for the variable *s*. The distribution of $T_s$ is obtained via random permutations of variable *s*, and the *p*-value elicited by the distribution of $T_s$ provides a measure for the relevance of variable *s* (algorithm details are in Tutz and Berger 2021); the proposed procedure, which is applied for each component (location and scale) is iterative, and it runs through the following steps:

- *step 0*: initialise via the model fit with category-specific intercepts only, and obtain the preliminary threshold estimates;
- *step 1*: consider the *s* variables and fit all the possible models with an extra split;
- *step 2*: select the best model as the one with the lowest *p*-value associated to the LR test-statistic;
- *step 3*: for each variable/split/component combination, do a permutation test using the maximal value statistic with a significance level $\alpha /{2s}$. If the observed value is significant, repeat from steps 1 to 3;
- *step 4*: fit the obtained final model.

### Models with category-specific effects

An alternative way to model heterogeneity is to let variables modify the thresholds via the location-shift models (Tutz and Berger 2017). In particular, the variance in the underlying continuous response does not vary across groups of individuals, yet the intercepts (thresholds) vary across the individuals. The location-shift approach is nested in the basic cumulative models when the proportional odds assumption is neglected and more flexibility is needed. That is, the linear predictor

$$\begin{aligned} \tau _{j}-{\varvec{X}}_{i}{\varvec{\beta }} \end{aligned}$$

in the basic models is replaced by the predictor

$$\begin{aligned} \tau _{j}-{\varvec{X}}_{i}{\varvec{\beta }_j} \end{aligned}$$

in which the effects of the *s*th variable, $\beta _{sj}$, depends on *j* and therefore may vary across categories. Of course, it is possible that only some of the variables have category-specific effects, whilst the remaining variables have the so-called *global effects*, that do not vary across categories. Extensions of the cumulative logit model with category-specific effects have been considered in the literature. The resulting non-proportional odds model and partial proportional odds model have been investigated extensively (see, for example Brant 1990; Peterson and Harrell Jr 1990; Bender and Grouven 1998; Cox 1995; Kim 2003; Liu et al 2009, among others).

The nested shift version of heterogeneity models uses the predictor

$$\begin{aligned} \tau _{j}-{\varvec{X}}_{i}({\varvec{\beta }+ {K}/{2}-j+1}) \varvec{\delta }, \end{aligned}$$

where $\varvec{\delta }$ indicates a *response style*, that is, a tendency to middle/extreme categories, and it explicitly models how variables change the subjects’ response behaviour: large $\varvec{\delta }$ and therefore more concentration in middle categories means smaller variation of responses, and small $\varvec{\delta }$, with more concentration in extreme categories, means higher dispersion.

As it is often the case, the increased flexibility improves the fit, at the expense of interpretation. In fact, the location-shift model, with category specific effects, comes with a much sparser parametrisation. For $K = 3$, the model with category-specific effects and the shift-version model are even equivalent. All in all, the main difference with the location-scale model is that the latter has a multiplicative structure (motivated by variance heterogeneity in the underlying continuous response) that yields to the dispersion effect; the location-shift model, instead, has an additive structure (motivated by the shifting of thresholds) that models the tendency to middle or extreme categories.

## DL perception analysis

**Fig. 1:**
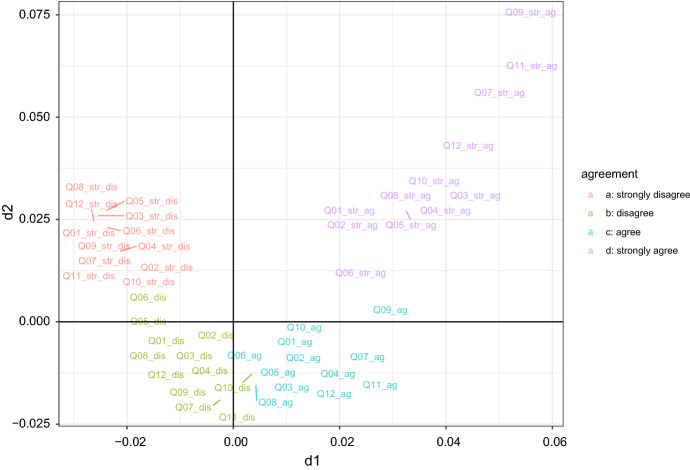
Variables map: the levels of agreement are, for all items, grouped together, and the different groups of levels are ordered from the top left side of the map (strongly disagree) till the top right side of the map (strongly agree): the variables pattern follows the *arch* effect, typical of CA solutions

**Fig. 2:**
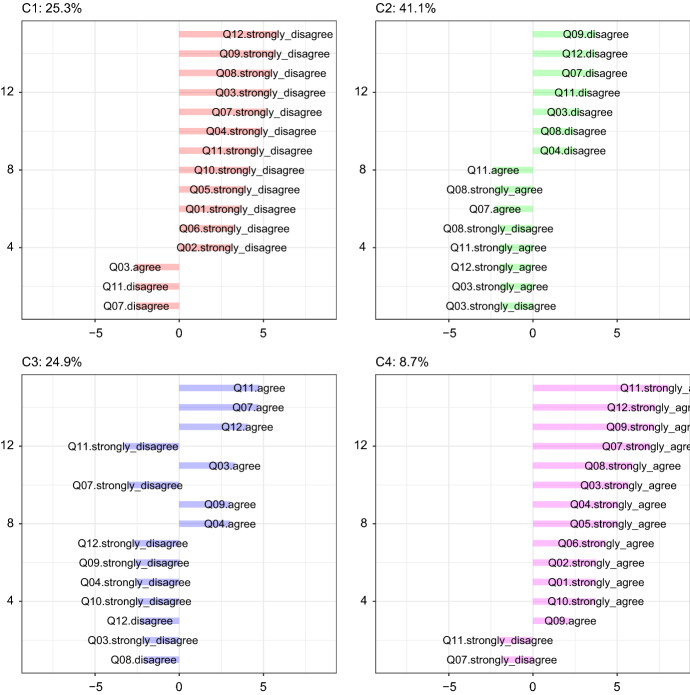
Item scores for groups characterisation: deviations from independence condition

The JDR step of the proposed hybrid approach is the CCA of DL perception-related items. The CCA hyper-parameter *K* is set to four, as high as the number of levels of each considered item. It is worth to remind that the items are statements indicating a positive perception of the DL experience. The variables (items) factorial map is depicted in Fig. 1; note that two dimensions are considered, even though the items come from a unidimensional scale: this is done for illustrative purposes, to provide a graphical representation to support the interpretation of the clustering solution. In fact, as in correspondence analysis (CA, see e.g., Greenacre 2007), variables levels are close to one another on the map if they have been selected by (almost) the same respondents: therefore, it is easily seen that similar levels of agreement/disagreement are grouped on the map. Furthermore, Fig. 1 shows the so-called *arch* or *Guttman effect*: the horizontal axis on the map separates disagreement from the agreement. The vertical axis separates the middle categories from the extreme ones. The arch effect occurs when a single numeric latent variable is dominant (see, e.g., Lebart and Saporta 2014): it underlies the variable levels and, as a consequence, the respondents. Therefore, the cluster solution is expected to identify groups of respondents with a similar attitude towards DL. This is confirmed by the cluster characterisation plot depicted in Fig. 2. Each barplot in the figure refers to a different cluster: the bars indicate the deviations from independence as described in Sect. 4. Since the obtained clusters consist of respondents that share similar levels of agreement/disagreement, it is fairly natural to sort out the clusters according to the predominant level of agreement and define the cluster allocation as the ordinal variable *Y*, with levels coded as 0, 1, 2, 3.

**Fig. 3:**
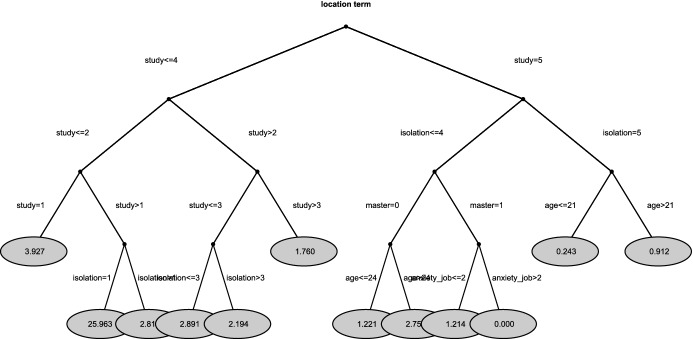
Tree for location term of DL data. The parameter estimates ${\hat{\beta }}_s$ are given in the terminal nodes

**Fig. 4:**
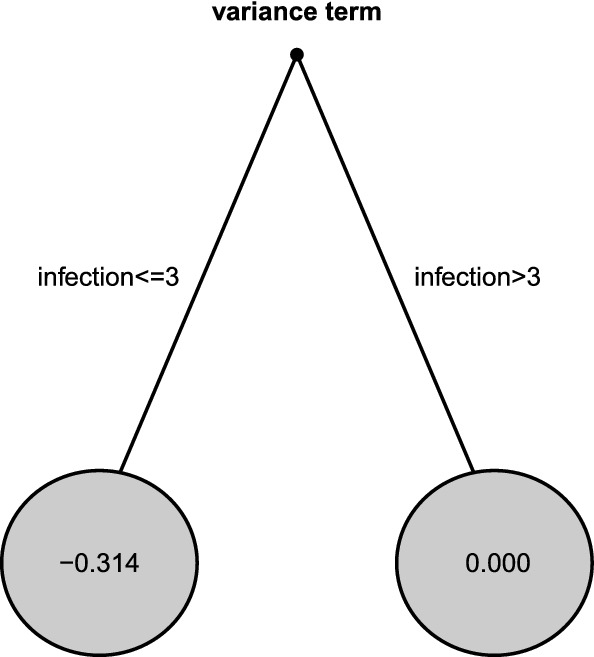
Tree for variance term of DL data. The parameter estimates $\varvec{{\hat{\eta }}}_s$ are given in the terminal nodes

To identify the main drivers of DL perception, we apply the location-scale recursive partitioning; Fig. 3 shows that most of the drivers are stress-related. Specifically, *study* (stress due to studying experience during the pandemic), *isolation* (stress due to the condition of social isolation), and *age* are the three variables which affect the location component. The most negative DL perception is found in the node where *study* $=5$: these respondents felt really stressed out by the studying experience during the pandemic. The respondents that felt less stressed about the studying experience, perceived high stress of isolation (*isolation* $=5$). However, students below 21 years of age had less severe stress of isolation ($\hat{\beta }_{Age \le 21}=0.243$), compared to students above 21 ($\hat{\beta }_{Age>21}=0.912$). Among students who comparatively perceive less stress of isolation, we found master students with ‘anxiety for employment because the salary would probably not be as excellent as they wish for the devastating effect of Covid-19’ (the measurement item derives by the anxiety scale in Mahmud et al 2021). The scale term-related tree in Fig. 4, indicates the variable *infection* (stress induced by the fear of contagion) as the main driver. In other words, students that indicated values higher than 3 the variable *infection* are more heterogeneous than students who perceived a lower risk of *infection*.

To further evaluate these issues we fitted the location-scale model described in Sect. 5. We selected the logit link function for simplicity as reported in Sect. 5 and after the inspection of the BIC index in Table 2.

The fitted model is

$$\begin{aligned} Y^{\star }_i= & {} -\underset{(0.113)}{0.591} \, study_{i} -\underset{(0.005)}{0.231} \, isolation_{i} +\\&+\underset{(0.046)}{0.171} \, infection_{i}+ \underset{(0.035)}{0.055 } \, anxiety_{i}+\\&+\underset{(0.040)}{0.135} \, age_{i} + \,{\hat{\sigma }}_i {\hat{\epsilon _i}}, \end{aligned}$$

with $i=1,\ldots , n$. The estimated thresholds are

$$\begin{aligned} {\hat{\tau }}_1= & {} -2.508 (0.531)\\ {\hat{\tau }}_2= & {} -1.018 (0.365)\\ {\hat{\tau }}_3= & {} 0.432 (0.330). \end{aligned}$$

Finally,

$$\begin{aligned} \log (\hat{\sigma }_i)= & {} -\underset{(0.2166)}{0.526} \, infection_{SS_i} -\underset{(0.196)}{0.398} \, infection_{MS_i}\\&-\underset{(0.194)}{0.539} \, infection_{VS_i} -\underset{(0.196)}{0.235} \, infection_{ES_i}, \end{aligned}$$

where *SS* stems for ‘somewhat stressful’, *MS* represents ‘moderately stressful’, *VS* ‘very stressful’ and *ES* ‘extremely stressful’. Note that the *master* variable has been dropped in the estimated location-scale model because the parameter related to that variable results to be no different from zero.

**Table 2:** Log-likelihood and BIC indexes for the different link functions (the smallest BIC value is in boldface)

Link	logLik	*BIC*
Logit	$-1735.70$	$\varvec{3495.401}$
Probit	$-1735.91$	3495.831
Log-log	$-1756.41$	3536.814
cLog-log	$-1772.19$	3568.391

DL positive perception increases with *age*, stress for *infection* and *anxiety*, and globally reduces for high levels of perceived stress both for studying experience and isolation. Results related to $\hat{\sigma }_i$, leading to different scales of the latent variable, highlight that heterogeneity increases for reduced levels of contagion-related stress.

The location-scale model is compared with the nested cumulative one with proportional assumption. The absence of heterogeneity effects (which implies $\sigma = 0$) has been formally tested via the LR-test. The value of the test statistics and the corresponding *p*-value of the test are 25.560 and $<0.001$, respectively, so that the null hypothesis of scale parameter equals to zero is rejected.

A further examination which confirms the validity of the proportional assumption has been made with the LR-test between cumulative models with (cumulative PA) and without (cumulative NPA) proportional assumption. The test statistic is 43.588 and the *p*-value of the test is $<0.001$ (BIC index of the model with non-proportional assumption is 3586.048 whereas 3555.927 is the BIC index for the model with parallel assumption) confirming the validity of the proportional assumption.

**Fig. 5:**
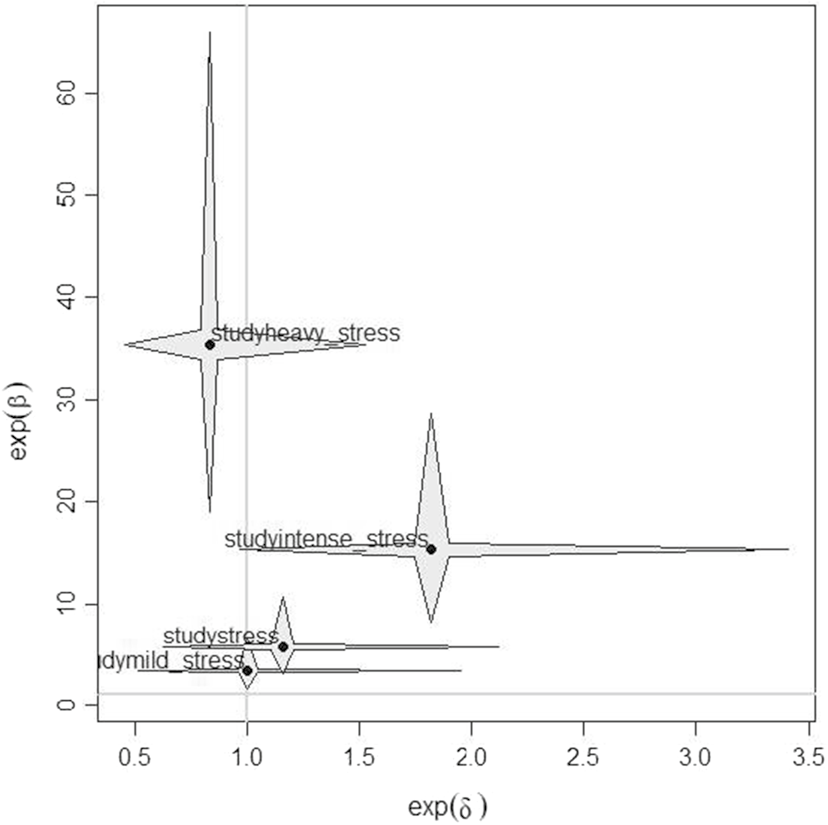
Effect stars for location-shift model with the only *study* variable

For the sake of completeness, the location-shift model is also computed on the same data with the selected by tree variables. The BIC index is 3638.99; in this case, several estimated parameters are not statistically significant with the only exception of *study*. A further examination of the only *study* variable on both parameters of the predictor highlights the role of the only ‘intense stress’ category. A visualization of the parameter estimates is reported in the star plot (Fig. 5); it shows the tuples $(\exp (\varvec{\hat{\delta }}),\exp (\varvec{\hat{\beta }}))$ for the linear effects of the location-shift model. The first value, $\exp (\varvec{\hat{\delta }})$, represents the heterogeneity effect on the odds, for values larger than one there is a tendency to middle categories, for values smaller than one there is a stronger tendency to extreme categories than in the simple proportional odds model. Thus, students reporting intense study stress concentrate in the central categories their DL perception (BIC index of this estimated model is 3620.887).

Summary results concerning BIC indexes of the alternative models reported in Sect. 5.2 and based on the same set of variables (selected by tree) are in Table 3.

**Table 3:** Log-likelihood and BIC indexes for the alternative models (the smallest BIC value is in boldface)

Models	logLik	*BIC*
Cumulative NPA	$-1726.686$	3586.048
Cumulative PA	$$-1748.48 $$	$$\varvec{ 3555.927}$$
Location-shift	$-1734.73$	3638.990

## Concluding remarks

The study proposes a hybrid method to analyse complex survey structures. The well-established synthesis by aggregation of the items from a same psychometric scale is a viable option, yet it inherently assigns the same importance to each item. The proposed hybrid approach defines data-driven weights for the item levels: the weighting system takes into account both the association structure of the items and the heterogeneity of the respondents. Furthermore, it is common practice, e.g., in path models (Jöreskog 1969), to study the effects dependency among subsets (blocks) of the considered items. In the same spirit, yet with an alternative approach, we synthesize the DL related subset of items, define a synthetic ordinal response, and then regress it on the other items according to appropriate models for ordinal data.

Among the latter, we moved in the context of cumulative data models, which represent the natural candidates when a latent trait is taken into account. In this area category-specific effects, which were treated extensively in the literature, can often be replaced by much simpler models that contain an heterogeneity term yielding much simpler and easy to interpret models. The latter typically provide better fit to the data and additional information on the effects of explanatory variables. If they are ignored, estimates may be biased.

In summary, once the synthetic ordinal response is obtained, a location-scale model taking account possible heterogeneity is applied, and a recursive partitioning-based variable selection method is used to identify the variables that affect the ordinal response and, indirectly, the items subset of interest (in this case, the DL-related items). The code and the pre-processed data are available on GitHub^1^.

Alternative models for ordinal data taking into account the proportional assumption and an additive structure motivated by the shifting of thresholds are also tested and compared in terms of global fitting showing worse off results with respect to the selected location-scale model. The latter is also implemented for the accuracy/completeness of the information on the ordinal variable obtained by discretising the continuous variable elicited summing up the twelve ratings of the DL scale. The discretisation with four equidistant thresholds, as reported in Ramsay (1973), yielded an ordinal variable on which the set of variables selected in the trees are regressed; a BIC index of 4094.075 highlighted worse fitting results than the proposed approach.

Empirical results underline some findings of the literature; the significant effect of stress and risk perception was consistent with previous studies addressing the psychological consequences of the Covid-19 pandemic on students’ lives and responses to distance learning (Aristovnik et al 2020; Bork-Hüffer et al 2021; Capone et al 2020; Unger and Meiran 2020). Specifically, students with stress due to social isolation and with stress due to academic life in remote are less satisfied and perceive a low feeling with respect to DL. On the opposite, having a high perceived risk for Covid-19 contagion increases the DL feeling and reduce the heterogeneity in the clusters of respondents (see also Bacci et al (2022)). Furthermore, previous studies point out differences in learning style according to student age (Chyung 2007; Dibiase and Kidwai 2010; Raidal and Volet 2009; Vermunt and Vermetten 2004). The literature indicates that older students spend more time on course related learning, spend more time using asynchronous learning tools, and report that they have very positive learning experiences in online courses as detected in our findings.

Limitation of the analysis concerns the sample design of the survey collected by means of a *chain sampling*, leading to an ‘observational study’.

Future work will refer to methodological and applied perspectives. From a methodological perspective, conditional models may be also analysed, albeit preliminary results by Iannario et al (2022) where mixture models with uncertainty (see Tutz 2020a) have been tested on the same DL data produced poor fitting results. Furthermore, we constrained ourselves to define a meta-item with the same number of levels as any other item in the survey. This is a sound choice given the survey at hand as the items all have the same four element scale. It is worth to note that the hybrid method can be rendered more flexible by allowing the meta-item to have a data driven number of levels: in doing so, however, one has to pick a suitable metric to evaluate the JDR clustering solutions and select the optimal number of clusters. From an application perspective, the complex survey can be further enhanced by considering a multilevel structure dictated by respondents demographics; furthermore, a similar survey can be administered to investigate students perception of blended learning, a combination of distance learning and classroom learning.

## Footnotes

1 https://github.com/alfonsoIodiceDE/hybrid_analysis_complex_categorical.
